# Foreign *cry1Ac* gene integration and endogenous borer stress-related genes synergistically improve insect resistance in sugarcane

**DOI:** 10.1186/s12870-018-1536-6

**Published:** 2018-12-10

**Authors:** Dinggang Zhou, Xiaolan Liu, Shiwu Gao, Jinlong Guo, Yachun Su, Hui Ling, Chunfeng Wang, Zhu Li, Liping Xu, Youxiong Que

**Affiliations:** 1 Key Laboratory of Sugarcane Biology and Genetic Breeding, Fujian Agriculture and Forestry University, Ministry of Agriculture, Fuzhou, 350002 Fujian China; 2 Key Laboratory of Ecological Remediation and Safe Utilization of Heavy Metal-Polluted Soils, Hunan University of Science and Technology, School of Life Science, Xiangtan, 411201 Hunan China

**Keywords:** Sugarcane, *cry1Ac* gene, Insect resistance, RNA-Seq, Transcriptome analysis, Phenotypic traits

## Abstract

**Background:**

Sugarcane (*Saccharum* spp. hybrids) is considered the most globally important sugar-producing crop and raw material for biofuel. Insect attack is a major issue in sugarcane cultivation, resulting in yield losses and sucrose content reductions. Stem borer (*Diatraea saccharalis* F.) causes serious yield losses in sugarcane worldwide. However, insect-resistant germplasms for sugarcane are not available in any collections all over the world, and the molecular mechanism of insect resistance has not been elucidated. In this study, *cry1Ac* transgenic sugarcane lines were obtained and the biological characteristics and transgene dosage effect were investigated and a global exploration of gene expression by transcriptome analysis was performed.

**Results:**

The transgene copies of foreign c*ry1Ac* were variable and random. The correlation between the cry1Ac protein and *cry1Ac* gene copies differed between the transgenic lines from FN15 and ROC22. The medium copy lines from FN15 showed a significant linear relationship, while ROC22 showed no definite dosage effect. The transgenic lines with medium copies of *cry1Ac* showed an elite phenotype. Transcriptome analysis by RNA sequencing indicated that up/down regulated differentially expressed genes were abundant among the *cry1Ac* sugarcane lines and the receptor variety. Foreign *cry1Ac* gene and endogenous borer stress-related genes may have a synergistic effect. Three lines, namely, A1, A5, and A6, were selected for their excellent stem borer resistance and phenotypic traits and are expected to be used directly as cultivars or crossing parents for sugarcane borer resistance breeding.

**Conclusions:**

*Cry1Ac* gene integration dramatically improved sugarcane insect resistance. The elite transgenic offspring contained medium transgene copies. Foreign *cry1Ac* gene integration and endogenous borer stress-related genes may have a synergistic effect on sugarcane insect resistance improvement.

**Electronic supplementary material:**

The online version of this article (10.1186/s12870-018-1536-6) contains supplementary material, which is available to authorized users.

## Background

Sugarcane (*Saccharum* spp*.* hybrids) is considered the most important crop for sugar production globally and is a valued raw material for the biofuel industry [1, 2]. Insect attack is a major issue in sugarcane cultivation, resulting in yield losses and sucrose content reduction [2]. One significant sugarcane pest is stem borer (*Diatraea saccharalis* F., Lepidoptera, Crambridae), which affects sugarcane throughout the entire growing season and causes serious yield losses of nearly 25–30% [3, 4]. However, insect-resistant sugarcane germplasms are not available in any collections [3]. In addition, modern sugarcane cultivars are highly complex polyploid-aneuploids, with chromosome numbers ranging from 80 to 130 [5]. It is thus almost impossible to breed an insect-resistant variety by means of traditional cross-breeding. Genetic engineering is expected to play an important role in improving the insect resistance of sugarcane [6–8] and could facilitate the development of insect-resistant sugarcane varieties or germplasms for use in cross-breeding.

To date, the *cry* gene has been effectively used to control stem-borer pests in many crops, including rice (*Oryza sativa*) [9], corn (*Zea mays*) [10], cotton (*Gossypium hirsutum*) [11], potato (*Solanum tuberosum*) [12] and soybean (*Glycine max*) [13], which have widespread commercial applications and verified safety [14–16]. There is an urgent need for borer resistance traits, and a series of studies that introduced the *cry* gene into sugarcane successfully obtained insect-resistant sugarcane lines [1, 4, 17–19]. However, only one case involving insect-resistant transgenic sugarcane has been approved for commercial planting in Brazil. The first report on insect-resistant sugarcane lines based on the use of the *cry1Ab* gene [20]. Recently, Gao et al. [18] successfully introduced the *cry1Ac* gene into sugarcane and obtained insect- resistant transgenic lines. Wang et al. [19] successfully introduced the *cry1Ab* and *EPSPS* genes together and obtained transgenic sugarcane lines with insect resistance and herbicide tolerance.

The influence of transgene copy number on gene expression levels is complex [21]. The gene balance hypothesis suggests that increasing the transgene copy number would upregulate gene expression levels, and thus a correlation (positive or negative) must exist between gene copy number and gene expression level [22]. Therefore, transcript abundance must increase with gene dosage in order to increase protein abundance [22]. However, low copy-number exogenous genes are considered to be beneficial for plant improvement, particularly in diploid plants [21]. It is generally considered that a low gene copy number would decrease the possibility of transgene co-suppression, while multiple gene copies may result in gene silencing and co-suppression [21]. To date, very little is known about the influence of the copy number of the foreign *cry1Ac* gene on its expression level in transgenic sugarcane via particle bombardment, apart from the findings of Joyce et al. [23], who suggested that transgene copy number does not influence the gene expression in transgene lines.

The objective of breeding is to obtain excellent agronomic characters or to retain the agronomic characteristics of the parental cultivar [24]. Thus, assessing the agronomic performance of transgenic sugarcane under field conditions is necessary in order to select excellent transgenic offspring [23]. Arencibia et al. [25] were the first to perform field trials of five insect-resistant transgenic sugarcane events and they found that most of the transgenic lines had agronomic traits similar to that of the receptor variety. Gao et al. [18] tested the field performance of transgenic *cry1Ac* sugarcane and discovered that these lines exhibited better phenotypic traits than the non-transgenic sugarcane. Wang et al. [19] investigated the field performance of five single-copy transgenic sugarcane lines, which exhibited excellent cane borer resistance and herbicide tolerance but poor agronomic traits.

The genetic engineering of plants with enhanced tolerance to biotic stresses (i.e., insect stress) typically involves complex multigene networks and may therefore have the potential to introduce unintended effects due to the location effect of transgene integration [26]. However, the *cry1Ac* transgenic sugarcane insertion site and its flanking sequence are complex, and the effects of particle bombardment remain unclear. The growth and development of plants, including transgenic plants, along with plant responses to abiotic and biotic stresses, involves a complex network of gene regulation [27]. In recent years, there has been an increasing number of reports on gene expression analysis in plants during development and stress using a global transcriptomic approach [27]. However, these reports mainly focused on model plants due to their available genome sequence information. Many studies have investigated the transcriptomes of non-model plants, including sugarcane, for which the sequencing of the whole genome is currently in progress [27]. Microarrays, serial analysis of gene expression and RNA sequencing (RNA-Seq) are the three most commonly used methods for transcriptome analysis in gene expression studies [28, 29]. RNA-Seq, which allows for the near-complete characterization of transcriptomic events occurring in a specific tissue at a certain time, has been widely applied and proven particularly useful in non-model plants, including sugarcane [28, 30–32]. For transgenic plants, a number of studies to date have used transcriptome analysis to study gene expression or assess the impact of genetic engineering [29, 33–36]. Misra et al. [35] identified differentially expressed genes (DEGs) in *AtMYB12*-expressing transgenic tobacco lines by microarray transcriptome analysis. Nietzsche et al. [36] employed a global transcriptional profiling approach using microarray to identify transcriptional changes in *35S-STKR1 Arabidopsis* in order to interpret the observed phenotypic and metabolic changes. Cai et al. [37] employed RNA-seq to identify transcriptional changes in transgenic *ZmWRKY17 Arabidopsis* to reveal salt stress and abscisic acid (ABA) responsive genes in the ABA signaling pathway. Chung et al. [38] performed RNA-Seq to identify the direct target genes of the OsNAC proteins in transgenic *OsNAC* rice and to elucidate the molecular regulatory networks of the root architectures of RCc3:OsNACs for drought tolerance. Based on all the above, a global transcriptome analysis in *cry1Ac* transgenic sugarcane could help identify DEGs and elucidate the selected or the foreign gene networks that are associated with the performance of sugarcane traits, particularly insect resistance.

To improve the insect resistance of sugarcane, the *cry1Ac* gene was genetically engineered via particle bombardment in our previous work [2, 17, 18, 39]. In the present study, in order to elucidate how foreign *cry1Ac* gene integration and endogenous borer-stress-related genes contribute to insect resistance improvement in sugarcane, and to select elite insect-resistant *cry1Ac* transgenic sugarcane lines, the performance and molecular characteristics of these lines, including the stalk borer damage level, the main agronomic traits and the correlation between *cry1Ac* gene copy number and gene expression level, were investigated. The expression of the foreign *cry1Ac* gene and DEGs were then analyzed using a global transcriptome approach by high-throughput RNA-Seq. Furthermore, the synergistic effects of the foreign *cry1Ac* gene and endogenous borer-stress related genes, and those genes identified from the DEGs associated with borer resistance metabolism in sugarcane, were also discussed. The present study provides novel insights into the mechanisms of foreign *cry1Ac* gene integration and endogenous borer stress-related genes in insect resistance improvement in sugarcane.

## Results

### Transgene copies via particle bombardment are variable and random

To estimate the copy number of the foreign *cry1Ac* gene, a quantitative TaqMan real-time PCR method was established. This method was based on double-standard curves of *cry1Ac* and *CYC/APRT/P4H* gene, which were integrated into the multi-target recombined plasmid pG1AcAPC0229 (p1AAPC). We discovered that, for transgene copies estimation in sugarcane, the internal reference genes *CYC, APRT,* and *P4H* did not differ significantly, obtaining a high amplification efficiency between 0.95 and 1.05 [2].

**Fig. 1:**
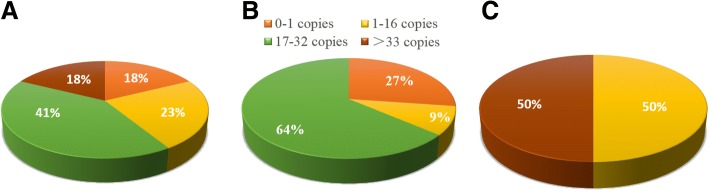
Proportion of different copy numbers of the *cry1Ac* gene in transgenic sugarcane. **a** Proportion of *cry1Ac* gene copy numbers in both FN15 and ROC22 transgenic sugarcane; **b** Proportion of *cry1Ac* gene copy numbers in FN15 transgenic sugarcane; **c** Proportion of *cry1Ac* gene copy numbers in ROC22 transgenic sugarcane

The *CYC* gene was selected for transgene copies estimation and the transgene *cry1Ac* copies in transgenic sugarcane are shown in Table 1. The proportions of different transgene *cry1Ac* copies in transgenic sugarcane are shown in Fig. 1. The cycle threshold (Ct) value of the endogenous reference gene *CYC* ranged from 23.837 ± 0.082~ 25.398 ± 0.029, and the mean Ct was 24.590 ± 0.076. Variance analysis indicated that there was no significant difference between the Ct value of all of the transgenic and control lines.

**Table 1:** Transgene copies of *cry1Ac* transgenic sugarcane by quantitative TaqMan real-time PCR

	Lines	*cry1Ac* Ct value	*cry1Ac* copies	*CYC* Ct value	*CYC* copies	Relative copies (*cry1Ac/CYC*)
Mean ± SE						
Group I	A1	26.25 ± 0.05	24.83 ± 0.77	24.10 ± 0.05^a^	54.23 ± 1.7	0.46 ± 0.02
	A2	26.21 ± 0.02	25.46 ± 0.20	24.10 ± 0.08^a^	51.79 ± 0.55	0.49 ± 0.01
	A3	26.13 ± 0.03	26.88 ± 0.64	24.37 ± 0.10^a^	45.12 ± 3.13	0.60 ± 0.02
	A4	26.24 ± 0.07	24.87 ± 1.20	24.37 ± 0.01^a^	45.16 ± 0.43	0.55 ± 0.05
	A5	26.04 ± 0.02	28.64 ± 0.40	24.28 ± 0.05a	48.02 ± 1.66	0.6 ± 0.01
	A6	26.19 ± 0.07	25.75 ± 1.27	24.52 ± 0.05^a^	40.62 ± 1.25	0.63 ± 0.05
	B1	35.99 ± 0.32	0.03 ± 0.01	24.45 ± 0.05^a^	42.66 ± 1.35	0.00 ± 0.00
	B2	34.94 ± 0.11	0.04 ± 0.00	24.33 ± 0.04^a^	46.35 ± 1.13	0.00 ± 0.00
	B4	35.07 ± 0.11	0.04 ± 0.00	23.84 ± 0.08^a^	65.22 ± 3.59	0.00 ± 0.00
	I2	27.59 ± 0.06	8.05 ± 0.32	24.70 ± 0.06^a^	35.92 ± 1.47	0.22 ± 0.02
	I4	26.00 ± 0.03	24.63 ± 0.57	24.76 ± 0.08^a^	34.48 ± 1.96	0.71 ± 0.03
Group II	D1	29.87 ± 0.04	1.59 ± 0.04	25.50 ± 0.01^a^	20.7 ± 0.07	0.08 ± 0.00
	D2	25.24 ± 0.06	35.60 ± 1.50	25.40 ± 0.03^a^	22.14 ± 0.44	1.61 ± 0.12
	D4	24.37 ± 0.01	63.47 ± 0.33	24.86 ± 0.01^a^	32.01 ± 0.19	1.98 ± 0.02
	K2	25.07 ± 0.01	39.78 ± 0.13	24.81 ± 0.02^a^	33.26 ± 0.35	2.39 ± 0.01
	K3	28.75 ± 0.027	3.37 ± 0.06	24.50 ± 0.03^a^	41.2 ± 0.91	0.08 ± 0.00
	K5	28.48 ± 0.02	4.04 ± 0.06	24.89 ± 0.07^a^	31.45 ± 1.43	0.13 ± 0.00
Control	FN15	–	–	24.60 ± 0.05^a^	38.54 ± 1.24	–
	ROC22	–	–	24.51 ± 0.03^a^	40.96 ± 0.87	–
	ddH_2_O	–	–	–	–	–

Note: *Mean* Mean value of three replicates, *SE* Standard error, “-” means undetected

For Group I (the transgenic lines from the receptor variety FN15), the copy number of the *cry1Ac* gene per single cell ranged from 0.03 ± 0.01 copies~ 28.64 ± 0.40 copies, of which the Ct value of the corresponding line was 35.987 ± 0.323 (B1) and 26.036 ± 0.02 (A5), respectively. For Group II (the transgenic lines from the receptor variety ROC22), the copy number of the *cry1Ac* gene per single cell ranged from 1.59 ± 0.04 copies~ 63.47 ± 0.33 copies, of which the Ct value of the corresponding line was 29.87 ± 0.04 (D1) and 24.37 ± 0.01 (D4), respectively.

Further analysis of the *cry1Ac* gene copies showed that 0–1 copies/2C accounted for 18%, while 1–16 (=16) copies/2C, 17–32 copies/2C, and > 33 copies/2C accounted for 23, 41, and 18%, respectively. For Group I, 0–1 copies/2C accounted for 27%, while 1–16 (=16) copies/2C and 17–32 copies/2C accounted for 9 and 64%, respectively. No line possessed a copy number of > 31 copies/2C. For Group II, 1–16 (=16) copies/2C and > 31 copies/2C accounted for 50 and 50%, respectively. No line possessed a copy number of 0–1 copies/2C and 11–30 copies/2C.

The results suggest that the copies of the exogenous *cry1Ac* gene in the transgenic sugarcane from particle bombardment are variable and random..

### Transgene expression showed varied relationships with transgene copies

The expression level of the cry1Ac protein in transgenic sugarcane is summarized in Table 2. Whether the transgenic lines were from the receptor FN15 or ROC22, significant differences were found among all transgenic lines (except B1 and B2) and between the transgenic and the control line. Among the Group I transgenic lines, the line with the highest cry1Ac protein expression was A3 with 547.45 ± 0.06 ng·g^− 1^, while for the Group II, K5 had the highest cry1Ac protein expression level of 113.42 ± 0.24 ng·g^− 1^.

**Table 2:** Cry1Ac protein content in sugarcane leaves detected by quantitative ELISA

	Lines	Cry1Ac protein (ng·g^−1^, Mean ± SE)	Lines	Cry1Ac protein (ng·g^−1^, Mean ± SE)
Group I	A1	445.79 ± 0.15^f^	B1	19.32 ± 0.18^i^
	A2	451.90 ± 0.24^e^	B2	19.45 ± 0.01^i^
	A3	547.45 ± 0.06^a^	B4	42.18 ± 0.21^h^
	A4	468.47 ± 0.02^d^	I2	90.99 ± 0.16^g^
	A5	501.78 ± 0.25^b^	I4	6.10 ± 0.05^j^
	A6	469.19 ± 0.08^c^		
Control variety	FN15	0.00 ± 0.05 k		
Group II	D1	102.56 ± 0.05^b^	K2	36.17 ± 0.23^f^
	D2	87.04 ± 0.35^c^	K3	24.23 ± 0.12^e^
	D4	67.3 ± 0.28^d^	K5	113.42 ± 0.24^a^
Control variety	ROC22	0.00 ± 0.03^g^		

Note: Lowercase in the column followed by the same letters mean no significant difference at *P* = 0.05 level to their corresponding receptor variety

**Fig. 2:**
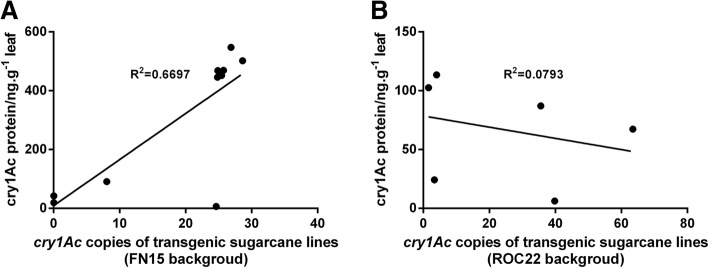
Correlation between *cry1Ac* gene copies and cry1Ac protein content in transgenic sugarcane. **a** Scatter diagram of the cry1Ac protein vs. *cry1Ac* gene copies for 11 *cry1Ac* transgenic lines from the receptor variety FN15: A1, A2, A3, A4, A5, A6, B1, B2, B4, I2, and I4; the *P*-value and R value from the Pearson’s correlation analysis were 0.002, 0.818, respectively. **b** Scatter diagram of the cry1Ac protein vs. *cry1Ac* gene copies for six *cry1Ac* transgenic lines from the receptor variety ROC22: D1, D2, D4, K2, K3 and K5. The *P*-value and R value from the Pearson’s correlation analysis were 0.589 and − 0.282, respectively

To explore the correlation between the cry1Ac protein and *cry1Ac* gene copies of the transgenic sugarcane, a scatter plot was drawn (Fig. 2). For Group I, especially, the A1–A6 lines with medium copies showed a significant linear relationship (*P*-value and R of the Pearson’s correlation analysis were 0.002 and 0.818, respectively), and the higher the *cry1Ac* gene copies, the higher the cry1Ac protein expression. Nevertheless, not all the medium-copy lines conformed to this linear relationship. For instance, the medium copy line I4 had a cry1Ac protein content of only 6.1 ± 0.05 ng·g^− 1^ leaf. In Group II, both the “high-copy” line D4 and “medium-copy” line K2 showed lower cry1Ac protein expression levels. For Group II, the *P*-value and R were 0.589 and − 0.282, respectively, which showed that no definite linear relationship existed between the cry1Ac protein and cry1Ac copies.

### *Cry1Ac* transgenic sugarcane exhibited excellent cane borer resistance

To investigate the insect resistance efficiency, the stalk borer damage level of the 17 *cry1Ac* transgenic lines and two receptor varieties FN15 and ROC22 are shown in Table 3.

**Table 3:** Variance analysis of borer damage ratio of *cry1Ac* transgenic sugarcane lines

	Lines	The borer damage percentage (%, Mean ± SE)	Lines	The borer damage percentage (%, Mean ± SE)
Group I	A1	15.00 ± 2.89^e^	B1	36.67 ± 3.33 ^c,d^
	A2	16.67 ± 1.67^e^	B2	40.00 ± 0.00 ^c,d^
	A3	11.67 ± 6.01^e^	B4	33.33 ± 6.67^d^
	A4	10.00 ± 0.00^e^	I2	46.67 ± 3.33^b,c^
	A5	8.33 ± 1.67^e^	I4	53.33 ± 3.33^b^
	A6	20.00 ± 5.00^e^		
Control variety	FN15	85.00 ± 2.89^a^		
Group II	D1	31.67 ± 1.67^c^	K2	36.67 ± 4.41^b,c^
	D2	33.33 ± 1.67^c^	K3	43.33 ± 1.67^b^
	D4	30.00 ± 2.89^c^	K5	33.33 ± 1.67^c^
Control variety	ROC22	93.33 ± 3.33^a^		

Note: Lowercase in the column followed by the same letters mean no significant difference at *P* = 0.05 level to their corresponding receptor variety

The results indicated that the borer damage percentage of the receptor varieties FN15 and ROC22 was as high as 85.00 ± 2.89 and 93.33 ± 3.33. For Group I, the borer damage ratio of all *cry1Ac* lines was significantly lower than that of the receptor variety FN15. In Group I, the damage ratio in lines A1–A6 was significantly lower than in B1, B2, B4, I2, and I4, and the line with the lowest borer damage ratio was A5 at only 8.33 ± 1.67. For the Group II transgenic lines, the borer damage ratio in all of the lines was lower than that of the receptor variety ROC22. These results indicated that the insect resistance efficiency of the *cry1Ac* transgenic lines of the receptor variety FN15 was better than that of ROC22. In addition, variance analysis indicated that there was no significant difference in the borer damage ratio between the receptor varieties FN15 and ROC22.

These findings showed that the introduction of the exogenous *cry1Ac* gene significantly improved the stem borer resistance of the transgenic sugarcane lines (Additional file 1: Figure S1, A1 line as an example), but the insect resistance effect of different receptor genotypes was variable.

### Phenotypic trait performance of *cry1Ac* transgenic sugarcane demonstrated commercial potential

Seventeen transgenic lines and two corresponding receptor varieties FN15 and ROC22, were subjected to major agronomic traits analysis (shown in Table 4).

**Table 4:** Performance of the major agronomic traits of *cry1Ac* transgenic sugarcane lines

	Lines	Diameter/cm	Height /cm	Brix values/%	Number of millable stalks/block	Sucrose content/%	Weight per stem Kg/stem	Sugarcane yield t/ha	Theoretical sugar yield t/ha
Group I	A1	3.14 ± 0.09^a,b^	233.1 ± 8.8^a^	21.48 ± 0.57^c,d^	240.0 ± 19.1^a^	15.22 ± 0.61^c,d^	1.80 ± 0.10^a^	138.79 ± 7.80^a^	21.13 ± 1.19^a^
	A2	2.99 ± 0.02^b,c^	214.5 ± 3.9^b^	20.71 ± 0.15^d^	216.0 ± 5.8^a,b,c^	14.72 ± 0.16^d^	1.51 ± 0.03^c,d^	104.22 ± 2.04^c^	15.34 ± 0.30^d^
	A3	2.80 ± 0.04 ^c,d^	205.8 ± 2.6^b^	20.61 ± 0.09^d^	222.7 ± 8.9^a,b^	14.61 ± 0.10^d^	1.27 ± 0.03^e^	90.39 ± 2.17^d^	13.20 ± 0.32^e^
	A4	2.97 ± 0.07^b,c^	196.9 ± 4.9^b^	22.09 ± 0.11^a,b,c^	207.6 ± 11.4^a,b,c^	16.21 ± 0.12^a,b,c^	1.36 ± 0.04^d,e^	90.71 ± 2.61^d^	14.70 ± 0.42^d^
	A5	3.12 ± 0.01^a,b^	213.7 ± 6.8^b^	22.55 ± 0.16^a^	220.0 ± 11.1^a,b^	16.71 ± 0.17^a^	1.63 ± 0.04^b,c^	115.13 ± 3.07^b^	19.24 ± 0.51^b^
	A6	3.08 ± 0.04^a,b^	203.9 ± 7.4^b^	22.04 ± 0.19^a,b,c^	217.2 ± 6.2^a,^^b,c^	16.16 ± 0.21a^b,c^	1.52 ± 0.05^c^	105.68 ± 3.53^b,c^	17.07 ± 0.57^c^
	B1	2.42 ± 0.02^f^	116.3 ± 2.1^d^	23.07 ± 0.15^a^	181.6 ± 18.4^c,d^	17.27 ± 0.16^a^	0.53 ± 0.00^g,h^	31.13 ± 0.16^e,f^	5.38 ± 0.03 ^f,g^
	B2	2.42 ± 0.09^f^	126.5 ± 5.5^d^	22.4 ± 0.21^a,b^	100.8 ± 6.4^f,g,h^	16.55 ± 0.23^a,b^	0.58 ± 0.05^f,g,h^	18.79 ± 1.62^g^	3.11 ± 0.27^i^
	B4	2.38 ± 0.06^f^	154.5 ± 9.3^c^	22.53 ± 0.19^a^	144.0 ± 6.0^e^	16.69 ± 0.20^a^	0.69 ± 0.07^f,g^	31.71 ± 3.24^e,f^	5.29 ± 0.54^f,g^
	I2	2.56 ± 0.08^e,f^	85.0 ± 7.83^e^	22.60 ± 0.35^a^	134.4 ± 13.8^e,f^	16.76 ± 0.38^a^	0.44 ± 0.03^h^	18.84 ± 1.33^g^	3.16 ± 0.22^i^
	I4	2.82 ± 0.10 ^c,d^	113.3 ± 4.7^d^	22.3 ± 0.42^a,b^	134.4 ± 12.4^e,f,g^	16.44 ± 0.45^a,b^	0.71 ± 0.05^f^	30.4 ± 2.21^e,f^	5.00 ± 0.36^g,h^
Control variety	FN15	3.25 ± 0.05^a^	209.9 ± 6.4^b^	21.47 ± 0.67^b,c,d^	206.4 ± 7.6^a,b,c^	15.54 ± 0.72^b,c,d^	1.74 ± 0.08^a,b^	115.15 ± 5.09^b^	17.89 ± 0.79^b,c^
Group II	D1	2.22 ± 0.03^b,c^	133.1 ± 7.3^e^	22.08 ± 0.17^b,c^	203.0 ± 6.7^a,^^b,c^	16.20 ± 0.18^b,c^	0.52 ± 0.01^c^	33.6 ± 0.92^d^	5.44 ± 0.15^e,f^
	D2	2.43 ± 0.08^b^	183.8 ± 8.6^c,d^	20.02 ± 0.31^e^	199.5 ± 3.6^a,b,c^	13.97 ± 0.33^e^	0.85 ± 0.08^b^	54.31 ± 5.34^b^	7.58 ± 0.75^b,c,d^
	D4	2.34 ± 0.08^b,c^	176.7 ± 5.5 ^c,d^	21.77 ± 0.17^b,c,d^	228.0 ± 12.9^a^	15.86 ± 0.18^b,c,d^	0.76 ± 0.04^b,c^	55.45 ± 3.04^b^	8.79 ± 0.48^b^
	K2	2.24 ± 0.07^b,c^	222.2 ± 6.5^a^	21.57 ± 0.38^b,c,d^	182.0 ± 13.5^c^	15.64 ± 0.42^b,c,d^	0.88 ± 0.07^b^	51.20 ± 4.31^b,c^	8.01 ± 0.67^b,c^
	K3	2.24 ± 0.05^b,c^	214.4 ± 7.6^a^	20.73 ± 0.43^d,e^	150.0 ± 9.3^d^	14.74 ± 0.47^d,e^	0.84 ± 0.04^b^	40.42 ± 1.94 ^c,d^	5.96 ± 0.29^d,e,f^
	K5	2.29 ± 0.06^b,c^	191.2 ± 7.9^b,c^	20.97 ± 0.28^c,d,e^	195.0 ± 5.6^b,c^	14.99 ± 0.31^c,d,e^	0.79 ± 0.02^b^	49.11 ± 1.17^b,c^	7.36 ± 0.18^b,c,d,e^
Control variety	ROC22	2.71 ± 0.07^a^	216.8 ± 6.5^a^	23.40 ± 0.20^a^	216.0 ± 5.3^a,b^	17.63 ± 0.22^a^	1.25 ± 0.09^a^	86.54 ± 6.29^a^	15.26 ± 1.11^a^

Note: Lowercase in the column followed by the same letters mean no significant difference at *P* = 0.05 level to their corresponding receptor variety

In Group I, the plant height of the transgenic line A1 (233.11 ± 8.76 cm) was significantly higher than that of FN15 (209.92 ± 6.35 cm), while those of lines A2, A3, A4, A5 and A6 did not differ significantly from that of FN15, ranging from196.89 ± 4.87 cm to 214.50 ± 3.89 cm. The remaining lines of Group I were significantly lower than that of FN15, with the height of line I2 being the smallest with an average height of only 85.00 ± 7.83 cm. In Group II, all of the lines had significantly lower heights than that of ROC22, except for lines K2 and K3. Stem diameter is an important agronomic trait in sugarcane. In Group I, the stem diameter of A1, A5, and A6 was similar to that of FN15 (no significant difference); however, the remaining transgenic lines in this group were significantly lower than that of FN15. In Group II, all of the lines had significantly lower stem diameters than that of ROC22. The Brix value reflects the sucrose content of sugarcane. The Brix value of most of the transgenic lines in Group I was equivalent to that of FN15, while the A5, B1, B4, and I2 lines were significantly higher than that of FN15. The probable reason is that stem borer damage significantly reduced the Brix value and sucrose content. The increased borer resistance of line A5 led to reduced damage and thus a higher Brix value, whereas the higher Brix values in lines B1 and I2 could be due to the decreased height and reduced diameter, which renders the storage capacity smaller and thereby increases the sucrose concentration. In Group II, the Brix value of each line was significantly lower than that of ROC22. As an important index of sugarcane yield, the number of productive tillers of lines B2, B4, I2, and I4 per block (31.2 m^2^) was significantly lower than that of FN15, while the other lines in this group were equivalent to that of FN15. The same pattern was found in the transgenic lines of Group II, except for line K2, which was significantly lower than that of ROC22.

### The theoretical sucrose yields varied in the *cry1Ac* transgenic sugarcane lines

Sugarcane yield and theoretical sucrose production are the two most important indexes of sugarcane as a crop. The sugarcane yield and theoretical sucrose yield results estimated from the plot survey are shown in Table 4. For transgenic line A1, the sugarcane yield and theoretical sucrose production (138.79 ± 7.80 t·ha^− 1^ and 21.13 ± 1.19 t·ha^− 1^) were significantly higher than that of FN15 (115.15 ± 5.09 t·ha^− 1^ and 17.89 ± 0.79 t·ha^− 1^). Furthermore, the sugarcane yield and theoretical sucrose production of lines A5 and A6 were equivalent to that of FN15, while those of the remaining lines were significantly lower than that of FN15 in this group. However, all of the transgenic lines of Group II were significantly lower than that of ROC22.

### Transcriptome dynamics in the *cry1Ac* transgenic sugarcane is demonstrated by RNA-Seq

Transcriptome analysis of the transgenic lines (A1, A5, and B4) and receptor variety FN15 was conducted using RNA-Seq technology.

Illumina sequencing of the 12 samples obtained 83.50 Gb clean reads with more than 95.48% Q20 bases and more than 90.67% Q30 bases for each sample (see Additional file 2: Table S1). The Trinity package assembled 65,995 unigenes, which was used as the sugarcane reference library, with an average length of 715 bp and N50 length of 1049 bp, including 13,813 unigenes measuring more than 1 kb (Additional file 2: Figure S2 and Table S2). A total of 65,995 unigenes were functionally annotated using the Non-redundant (Nr), Swiss-Prot, EuKaryotic Orthologous Groups (KOG), and Kyoto Encyclopedia of Genes and Genomes (KEGG) databases and the annotation statistics are shown in Additional file 2: Table S3.

The DEG statistics are shown in Additional file 2: Figure S3. A total of 6675 DEGs (4744/1931 up−/down-regulated), 9181 DEGs (6410/2771 up−/down-regulated), and 5050 DEGs (1330/3720 up−/down-regulated) were identified in transgenic lines A1, A5, and B4 compared with CK, respectively. The pathway enrichment analysis provided important information for investigating specific biological processes that could be influenced by the expression of the foreign *cry1Ac* gene in sugarcane.

**Fig. 3:**
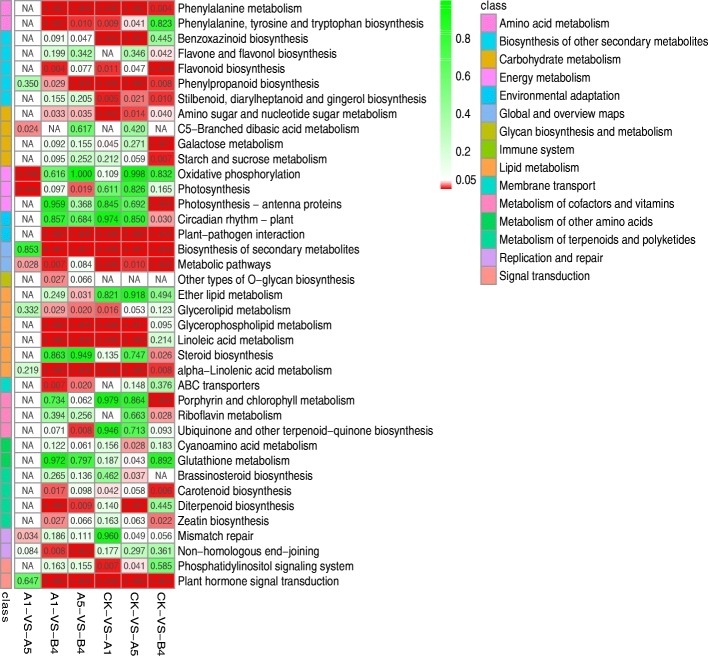
Heat map of the enriched KEGG pathways for differentially expressed genes. Pathway enrichment analysis was used for all the identified differentially expressed genes detected by RNA-Seq. The color bar on the left represents the different KEGG classification annotations. Each row represents a pathway (at least one sample of *P* ≤ 0.05), and each column represents a comparison group

The large number of DEGs allowed for functional annotation by KEGG enrichment analysis. Pathway enrichment analysis showed that the DEGs were mainly enriched in pathways associated with signal transduction, biosynthesis of secondary metabolism, environmental adaptation and lipid metabolism (Fig. 3). As shown in Fig. 3, plant hormone signal transduction, plant-pathogen interaction, benzoxazinoid biosynthesis, phenylpropanoid biosynthesis, and flavonoid biosynthesis were also regulated. These results are similar to those of previous studies whereby MAPKs (mitogen-activated protein kinases), Ca^2+^ channel proteins, ROS (reactive oxygen species), JAZs (jasmonate ZIM-domain proteins) and PAL (phenylalanine ammonia lyase) are involved in anti-insect related signaling pathways [40, 41].

In addition to these anti-insect metabolic pathways, some basic metabolic pathways, such as starch and sucrose metabolism, galactose metabolism (carbohydrate metabolism), and photosynthesis-antenna proteins (energy metabolism), were also altered. These are anticipated to affect the growth and development of plants, which would probably result in the poor performance of agronomic traits (i.e., short and slender stalks) of the transgenic line B4, while no effect on the growth and development of lines A1 and A5 was observed.

### The expression of endogenous borer-stress responsive genes in *cry1Ac* transgenic sugarcane is differentially regulated

To elucidate the molecular mechanisms of insect resistance, the differential expression of endogenous borer-stress responsive genes was investigated.

**Fig. 4:**
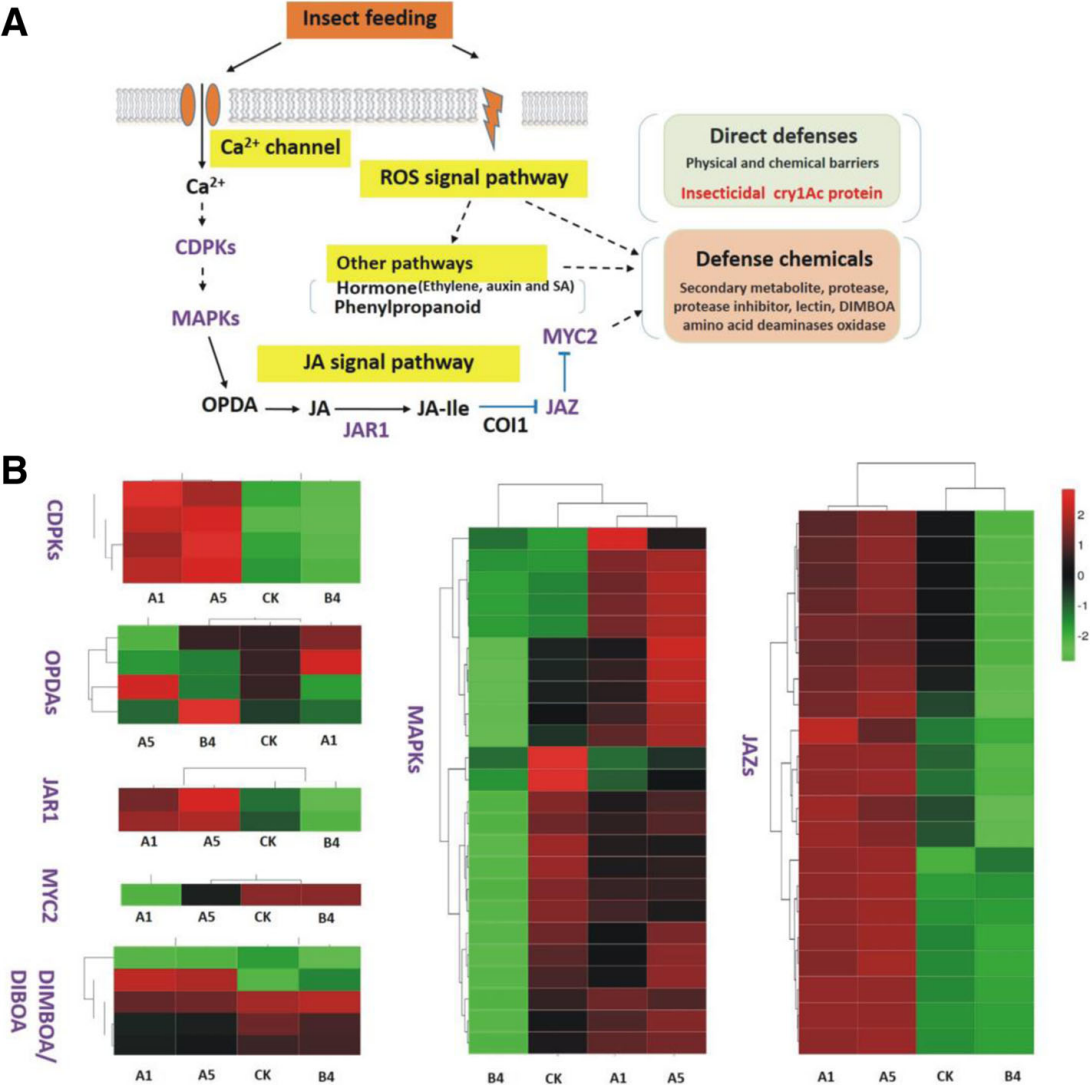
A model process of transgenic sugarcane responses to insect attack and heat maps of endogenous borer-stress related differentially expressed genes. **a** A model process of transgenic sugarcane responses to insect attack; **b** Heat maps of endogenous borer-stress related differentially expressed genes. CDPKs, OPDAs, MAPKs, JAR1, JAZs and MYC2: induced defense proteins related to insect attack; DIMBOA/DIBOA: hydroxamic acids, direct defense proteins related to insect attack

For Ca^2+^ channel-related proteins, group comparison analysis of the transgenic sugarcane and the control line (CK vs. A1) indicated that four DEGs encoding calcium-dependent protein kinases (CDPKs, all four DEGs were up–regulated) were observed, while three DEGs (all three DEGs up–regulated) in the group comparison of CK vs. A5, and zero DEGs in the group comparison CK vs. B4 were observed. Additionally, eight DEGs that encoding MAPKs (three up-regulated DEGs and five down-regulated DEGs) were observed in group comparison CK vs. A1, while 14 DEGs (nine up-regulated DEGs and five down-regulation DEGs) were observed in the group comparison of CK vs. A5, and 17 DEGs were observed in the group comparison of CK vs. B4 (all 17 DEGs were up–regulated) (Additional file 3: Table S4 and Fig. 4).

For JA signaling pathway related genes, all the DEGs encoding JAR1 (jasmonic acid resistant 1), JAZs and MYC2 (a basic-helix-loop-helix transcription factor) were observed to be up-regulated or down-regulated. All of the DEGs encoding these proteins in transgenic lines A1 and A5 were up-regulated compared to the non-transgenic sugarcane, while all of the DEGs encoding JAZs and MYC2 in the transgenic line B4 were down-regulated. For the group comparison of CK vs. A1, two DEGs encoding JAR1, 19 DEGs encoding JAZs, and one DEG encoding MYC2 were identified. Similarly, for the group comparison of CK vs. A5, two DEGs encoding JAR1, 19 DEGs encoding JAZs, and one DEG encoding MYC2 were identified. For the group comparison of CK vs. B4, though eight DEGs encoding JAZs and one DEG encoding MYC2 were identified, no DEG encoding JAR1 was observed.

For benzoxazinoid biosynthesis-related genes, group comparisons of CK vs. A1 indicated that four DEGs encoding hydroxamic acids (DIMBOA/DIBOA) biosynthetic enzymes (two up-regulated DEGs and two down-regulated DEGs) were observed, while five DEGs (two up-regulated DEGs and two down-regulated) in the group comparison of CK vs. A5, and only one down-regulated DEG in the group comparison CK vs. B4 were observed. (Additional file 3: Table S4 and Fig. 4).

The findings of these DEG analyses suggest that endogenous borer-stress related genes play a positive role in improving insect resistance in transgenic sugarcane lines A1 and A5, but negatively influence line B4.

## Discussion

Controlling diseases and pests is necessary for achieving food security. The use of resistant varieties is widely considered as one of the most cost-effective measures for achieving food security. Both traditional cross breeding and modern genetic engineering breeding approaches have been employed to improve crop resistance, productivity, and other desirable traits. Transgenic breeding, in particular has tremendous potential for the introduction of novel traits that are not normally present in the plant genome, i.e., herbicide tolerance or insect resistance [42–49].

Sugarcane borer damage causes great agricultural losses, resulting in approximately 25–30% losses in cane yield and 15% reductions in sucrose content annually [3, 4, 50]. However, traditional cross-breeding for sugarcane insect resistance is limited owing to both the lack of insect-resistant germplasms and the complexity of the inheritance background [3, 5]. Prior work has documented the effectiveness of transgenic crops expressing Bt insecticidal proteins. The cry1Ac protein has become an important tool in pest management in crops [51]. As an industrial and a vegetative propagation crop, sugarcane is suitable for insect-resistant transgenic modification via the importation of *Bt* genes such as *cry1Ac*. Therefore, several transgenic sugarcane lines expressing the cry1Ac crystal protein were developed in our previous study [18]. The main purpose of the present study was to explore how foreign *cry1Ac* gene integration and endogenous borer-stress related genes improve insect resistance in sugarcane, as well as to screen for elite insect-resistant transgenic *cry1Ac* lines.

Simple transgene integration enables molecular characterization [52]. Integration of a single and intact copy (or low copies, 1–2) of the transgene expression cassette can reduce the potential for unintended insertional inactivation events and also avoid the transgene silencing associated with complex integration events [52]. However, it is possible that the low copy number of the *cry1Ac* gene integrated into the sugarcane genome is ineffective for the improvement of insect resistance, while a high copy number of *cry1Ac* may result in deterioration of non-objective traits (i.e., yield and quality traits) by the energy consumption [2]. In the present study, the transgene copies of c*ry1Ac* in transgenic sugarcane were variable and random. This illustrated that transformation by particle bombardment produced transgenic sugarcane with complex integration events, such as variable copy numbers. The results support those observations in earlier studies wherein transgene integration is typically complicated by particle bombardment [52–54]. The exogenous *cry1Ac* gene was randomly inserted into various loci in the sugarcane nuclear genome. These results are also in agreement with Ismail’s [1] findings whereby independent transgenic sugarcane lines exhibited different copies of the *cry1Ac* gene, but harbored the same cassette of the foreign gene sequences and exhibited insect resistance [1].

One interesting finding in the present study is that the *cry1Ac* transgenic sugarcane lines with a medium copy number are favorable for the improvement of insect resistance traits and tend to increase the probability of obtaining the transgenic events. This finding is contrary to previous studies that suggested that transgenic diploid plants with relatively low copy foreign genes often exhibit stable integration and appropriate expression [53]. The main cause of this difference between the transgenic diploid plants and sugarcane is probably due to the complex ploidy (at least octoploid, chromosome number 120 or more) and large genome (up to 10 Gb) of the modern sugarcane cultivars. Differences between diploid plants and polyploid sugarcane may have influenced the transgenic traits such as insect resistance.

Another important finding is that different receptor varieties (FN15 or ROC22) showed different patterns with regards to the relationship between cry1Ac protein expression level and *cry1Ac* gene copy number in transgenic sugarcane. For Group I (FN15 background) lines, a significant linear relationship was found, although one line (I4) was an exception. The lines with medium copies (A1–A6) showed high levels of the cry1Ac protein, while lines with low copies (B1, B2, B4, and I2) showed much lower cry1Ac protein amounts. However, for Group II (ROC22 background), no obvious linear relationship was observed. For Group II, our findings are inconsistent with the dosage effect theory or the GBH theory, which suggests that the greater the transgene copy number, the higher its expression level [22, 55, 56]. Transgene copies can greatly affect expression levels [55, 56]. However, the insertion site bias of foreign genes in the genome of a receptor and the differences in the rearrangement of foreign genes by the nuclear genome could result from differences in germplasm resources, while the position of the foreign gene integration/insertion site and integrity of the expression frame of the foreign gene, can also have a profound impact on transgene expression [21, 56–58]. Theoretically, the foreign *cry1Ac* gene can integrate in the sugarcane genome at virtually any site and almost exclusively at random. The insertion sites of the foreign *cry1Ac* gene may influence transgene expression such that some integration may occur in higher or lower transcriptionally active chromatin and the surrounding endogenous regulatory sequences such as enhancers and inhibitors, or in condensed and even in transcriptionally inert chromatin regions [56, 58]. Transgenes in heterochromatic areas such as *cry1Ac* gene integration surrounding centromeres, are prone to silencing and give rise to reduced and/or variable expression. Therefore, it is possible to detect the cry1Ac protein expression in those lines (B1, B2, and B4) with very low cry1Ac copies (0~ 1 copies, nearly 0), though this is an unusual phenomenon according to the dosage effect theory. This suggests that a positive correlation exists between the cry1Ac protein expression level and the copies of *cry1Ac* gene, if the transgene copies and integration location are appropriate.

The present study also provides new information on the performance of *cry1Ac* transgenic sugarcane lines. The results showed that the *cry1Ac* transgenic sugarcane lines exhibited excellent borer resistance but demonstrated genotypic differences (Table 2 and Additional file 1: Figure S1). The findings further supported that *cry1Ac* gene integration, whether into diploid plants such as *O. sativa*, or into highly complex polyploid plants such as sugarcane, can tremendously improve the resistance to borer attack [9]. In terms of agronomic performance, the transgenic sugarcane line A1 had an average cane yield of 138.79 ± 7.80 t/ha, which was almost 1.3-fold higher than that of the control sugarcane plants (FN15). Furthermore, the yield and theoretical sucrose production of two lines (A5 and A6) were equivalent to that of FN15, while the lines from the receptor ROC22 were significantly lower than that of ROC22 (Table 4). These results indicated that *cry1Ac* gene integration increases the genetic diversity of the receptor sugarcane variety. Thus, the transgenic progenies were variable and the elite transgenic offspring were bred through a process of selection.

The current study also provided novel insights into the molecular mechanisms of insect resistance in *cry1Ac* transgenic sugarcane lines. The most interesting result is that the main altered pathways of the transgenic lines A1 and A5 were enriched in the plant hormone signal transduction, plant-pathogen interaction, benzoxazinoid biosynthesis, phenylpropanoid biosynthesis, flavonoid biosynthesis, and linoleic acid metabolism, which are involved in anti-insect related progresses, while the primary altered pathways of the transgenic line B4 differed from those in A1 and A5. During the process of plant insect defense, the main function of MAPKs is the transduction of extracellular stimuli into intracellular responses [59]. The Ca^2+^ channel and JA signaling pathway play a crucial role in the regulation of insect defense responses in plants [40, 41]. This study revealed that the DEGs identified by transcriptome analysis were enriched in these defense metabolic pathways, which corroborates the results of previous studies [40, 41]. The JA signaling pathway is considered to be the most important hormone signaling pathway for insect resistance [41]. All of the DEGs encoding JAR1, JAZs, and MYC2 in the JA signaling pathway were up-regulated in the medium-copy transgenic sugarcane lines A1 and A5, while all of the DEGs encoding JAZs and MYC2 were down-regulated in line B4. The differential expression of these JA-related genes may explain the relatively good correlation between improved insect resistance and medium copies for the transgenic sugarcane lines A1 and A5. In addition, the DEGs were mainly involved in plant hormone transduction pathways, phenylalanine metabolism, glycerophospholipid metabolism, linoleic acid metabolism, flavonoid biosynthesis and diterpenoid biosynthesis. These metabolic pathways have been classified as resistance induced pathways and play a key role in the defense against insect attack [60]. Thus, a synergistic effect of the foreign *cry1Ac* gene and the endogenous borer stress-related genes contributed to improved insect resistance in sugarcane. Further research should aim to investigate the precise function of these defense-related genes. A future study focusing on the defense metabolism pathways is therefore warranted.

## Conclusions

In this study, foreign *cry1Ac* gene integration dramatically improved insect resistance in transgenic sugarcane with medium *cry1Ac* gene copy numbers. RNA-Seq analysis revealed that up/down-regulated DEGs were abundant among the *cry1Ac* sugarcane lines and the receptor variety, and the foreign *cry1Ac* gene and endogenous borer stress-related genes may act synergistically. Three lines (A1, A5, and A6), exhibiting an improved phenotype in terms of yield and quality traits and a lower borer damage ratio were selected and are expected to be used directly as cultivars or crossing parents for sugarcane borer resistance breeding in the future.

## Methods

### Plant materials

Two receptor varieties (FN15 and ROC22) and 17 *cry1Ac* transgenic sugarcane lines (**Group I**, 11 lines from receptor variety FN15: A1, A2, A3, A4, A5, A6, B1, B2, B4, I2, and I4; **Group II**, six lines from receptor variety ROC22: D1, D2, D4, K2, K3 and K5) were nursed by the Key Laboratory of Sugarcane Biology and Genetic Breeding, Ministry of Agriculture/Fujian Agriculture and Forestry University, China. All of these transgenic sugarcane lines with the foreign *cry1Ac* gene were derived from particle bombardment [18].

### Estimation of foreign *cry1Ac* gene copy number and detection of protein expression

Fresh leaves from three individual plants from the same line, both for the *cry1Ac* transgenic lines and the receptor varieties, were sampled as biological replicates for the estimation of *cryAc* copies and determination of cry1Ac protein content. Total genomic DNA was extracted using a CTAB protocol described previously [17]. The DNA quality and integrity were assessed based on the A_260_/A_280_ ratio and electrophoresis. All of the DNA samples were stored at − 20 °C.

#### Foreign gene *cry1Ac* copy number

A quantitative TaqMan real-time PCR method was established to estimate *cry1Ac* gene copies. Three endogenous reference genes with potential low copy numbers, adenosine-5- phosphosulfate reductase (*APRT*), cyclin (*CYC*), and prolyl-4-hydroxylase (*P4H*) were cloned and a multi-endogenous reference gene standard plasmid (p1AcAPC) for transgene copy number identification was constructed [2, 61]. This plasmid p1AcAPC contained not only the three endogenous reference genes *APRT*, *P4H* and *CYC*, but also the foreign gene *cry1Ac* (see the Additional file 4: Figure S4). The primer sequences and TaqMan probe sequences for the real-time PCR are listed in Additional file 5: Table S5. Tenfold serial dilutions of the plasmid p1AcAPC with 1.0 × 10^8^ to 1.0 × 10^1^ copies per μL were prepared for assay. Sterile deionized water was used as blank control. Real-time PCR was performed using an ABI 7500 thermal cycler (Foster, USA). The reactions were performed using final volumes of 25.0 μL, including 12.5 μL of 2 × Fast Start Universal ProbeMasterMix, 1.0 μL template DNA (25.0 ng/μL diluted genomic DNA/plasmid), 1.0 μL (10 μmol/L) each of the forward and reverse primers and sterile ddH_2_O with the following program: 50 °C 2 min; 95 °C 10 min; 45 cycles of 95 °C 15 s, 60 °C 1 min. Each sample had three replications. The Ct values were obtained after the reaction. A standard curve (y = k × X + b) was established by plotting the Ct vs. the natural log of the copies. The total *cry1Ac* copies (10^Xt^) was calculated by relating the Ct value (Yt) to the corresponding standard curve. Then, the single cell copy number of each sample was calculated using the following formula [17]: copies/genome =10^Xt^/[25 n g × 10^− 9^ × 6.02 × 10^23^/ (10,000 (M bp) × 10^6^ × 660)]. The data are presented as mean values with standard error (SE), and one-way repeated measures analysis of variance (ANOVA) was used to test the differences in single cell copy number of the *cry1Ac* gene in the sugarcane samples. All of the analyses were conducted in EXCEL 2013 (Microsoft Inc., Redmond, WA, USA) and SPSS 11.5 statistical software (SPSS Inc., Chicago, IL, USA).

#### Cry1Ac protein expression

A QuantiPlate™ Kit for Cry1Ab/Cry1Ac (Envirologix, Inc., USA) was used to measure the cry1Ac protein content in the transgenic sugarcane leaves according to the manufacturer’s instructions. Each sample had three replications. Cry1Ac standards at concentrations of 0.15625, 0.3125, 0.625, 1.25, 2.5 and 5.0 ppb were used for calibration. OD_450_ values were measured with a microplate reader (Biotek, gene, USA). The data are presented as mean values with SE, and the cry1Ac protein concentration data were analyzed by one-way repeated measures ANOVA. Percentage data (protein concentration in the sugarcane leaves) were transformed using arcsine [square root (x)] prior to analysis. All of the analyses were conducted in EXCEL 2013 (Microsoft Inc., Redmond, WA, USA), SPSS 11.5 statistical software (SPSS Inc., Chicago, IL, USA) and GraphPad Prism 6 (GraphPad Prism Software Inc., San Diego, CA, USA).

### Evaluation of stalk borer damage level and phenotypic traits

The transgenic sugarcane lines and their receptor varieties were employed for phenotypic trait analysis. The phenotypic traits and stem border damage ratio of these transgenic sugarcane lines and their receptor were investigated in field trials carried out at the field station located at Fujian Agriculture and Forestry University.

In the isolated field in Fuzhou, which had been approved by the Ministry of Agriculture of China, the *cry1Ac* transgenic sugarcane lines and non-transgenic controls were evaluated from 2013 to 2014 using a randomized complete block design with three replications. Each block had three rows, and each row was 8 m in length with 1.3 m spacing between the rows. Each block covered an area of 31.2 m^2^. All the sugarcane plants were cut into single bud setts and were planted with 15 single bud setts per meter. During the harvest season in January 2014, phenotypic traits were investigated. Stem diameter (about 1 meter above the base of the stalk), stalk height (from the base of the stalk to the first visible dewlap), and Brix values were measured in 10 consecutive principal stalks in each block. The number of millable stalks and the theoretical cane yield per hectare were calculated based on the area, number and weight of the stalks, and the theoretical sucrose yield was calculated based on the average cane yield and sucrose content using equations as described by Wang et al. [19]. Twenty individual plants in each block were randomly measured to investigate the stalk borer damage. Stalk borer damage level was calculated according to the percentage of stalks damaged by the borer in all of the stalks. The results were expressed as the mean values ±SE of three replicates. Statistical analyses were performed by Microsoft Excel 2013 and SPSS11.5 (SPSS Inc., Chicago, IL, USA) using Student’s t test and one way ANOVA.

### Transcriptome analysis

According to the copy number estimation results, three *cry1Ac* transgenic sugarcane lines and the non-transgenic sugarcane line FN15 (CK) were selected for RNA-Seq. After inoculating with *D. saccharalis*, the youngest fully-expanded leaf (namely + 1 leaf) was collected from these four sugarcane lines, which were nursed in the greenhouse of the Key Laboratory of Sugarcane Biology and Genetic Breeding, Ministry of Agriculture/Fujian Agriculture and Forestry University, China. Five leaves were collected randomly from five individual plants of each sugarcane line, and combined as one biological replicate. Three biological replicates were assessed for each line. All of the samples were frozen in liquid nitrogen immediately after sampling and stored at − 80 °C until total RNA extraction. Total RNA was isolated using the TRIzol Reagent Kit (Invitrogen, Carlsbad, CA, USA) following by the manufacturer’s instructions. Ten micrograms of high quality RNA was used for the RNA-Seq analysis (Illumina HiSeq™ 2500/PE125).

Transcriptome sequencing, de novo assembly, evaluation and functional annotation were performed by Gene Denovo Co., Guangzhou, China. All of the sequencing reads were deposited in the National Center for Biotechnology Information under the Bioproject number PRJNA436063 with the Sequence Read Archive (SRA) accession number SRP133796. Data filtering, de novo assembly of the clean reads, and functional annotation were conducted as previously described [28]. Clean reads were obtained using a perl script by removing poly-A tails, adaptors and contaminants. The clean reads from each sample were hen merged and de novo assembled by the Trinity Program as a reference library, due to absence of a sugarcane reference genome [28, 62]. Function annotation was achieved by searching the unigenes against the Nr, COG (Clusters of Orthologous Groups of proteins database), KEGG and Swiss-Prot [63]. Gene Ontology (GO) annotation and functional classification were performed using Blast2GO and WEGO, respectively.

DEGs between the *cry1Ac* gene “medium-copy” group (*n* = 3) and “low-copy” group (n = 3) were analyzed using EdgeR [64]. Reads per kilobase per million reads (RPKM) values were used to normalize gene expression levels. Genes with more than 2-fold change (log2FC ≥ 1) with *P*-value ≤0.01 and false discovery rate (FDR) ≤ 0.05 were classed as DEGs in this study. KEGG enrichment analysis was used to dissect the molecular mechanisms of borer resistance according to a method described previously [65]. After using the log10(RPKM) values of the DEGs for normalization, a heat map was performed using the heat map illustrator software HemI 1.0.3.7 (http://hemi.biocuckoo.org/).

## Additional files

Additional file 1: **Figure S1.** Insect-resistant phenotype of the *cry1Ac* sugarcane and the receptor variety FN15 (CK). (PDF 314 kb)

Additional file 2: Supplementary RNA-Seq data file, including **Table S1**, **Table S2**, **Table S3**, **Figure S2** and **Figure S3** **Table S1.** The base pair information of high quality clean reads for de novo assembly. **Table S2.** Assembly statistics of the sugarcane unigenes. **Table S3.** Unigenes basic annotation statistics with Nr, Swiss-Prot, KOG and KEGG. **Figure S2.** The length distribution of sugarcane unigenes. **Figure S3.** Statistics of differentially expressed genes. (PDF 56 kb)

Additional file 3: **Table S4.** Differentially expressed unigenes involved in borer-stress responsive pathway. (XLSX 21 kb)

Additional file 4: **Figure S4.** Construction of the plasmid pG1AcAPC (p1AcAPC). (PDF 148 kb)

Additional file 5: **Table S5.** The primer sequences and TaqMan probe sequences for real-time PCR. (DOCX 12 kb)

## Abbreviations

*APRT*: Adenosine-5- phosphosulfate reductase
CDPK: Calcium-dependent protein kinase
*CYC*: Cyclin
DEGs: Differentially expressed genes
DIMBOA/DIBOA: Hydroxamic acids
FDR: False discovery rate
JAR1: Jasmonic acid resistant 1
JAZs: Jasmonate ZIM-domain proteins
KEGG: Kyoto encyclopedia of genes and genomes
MAPK: Mitogen-activated protein kinase
MYC2: A basic-helix-loop-helix transcription factor
*P4H*: Prolyl-4-hydroxylase
PAL: Phenylalanine ammonia lyase
ROS: Reactive oxygen species
RPKM: Reads per kilobase per million reads
