# Supplementary material for: Foreign cry1Ac gene integration and endogenous borer stress-related genes synergistically improve insect resistance in sugarcane
Source: BMC Plant Biol. 2018 Dec 10;18:342. doi: 10.1186/s12870-018-1536-6 (PMC6288918; doi:10.1186/s12870-018-1536-6)
Supplement: Supplementary file 1 — Figure S1. Insect-resistant phenotype of the cry1Ac sugarcane and the receptor variety FN15 (CK). (PDF 314 kb) [file 12870_2018_1536_MOESM1_ESM.pdf]

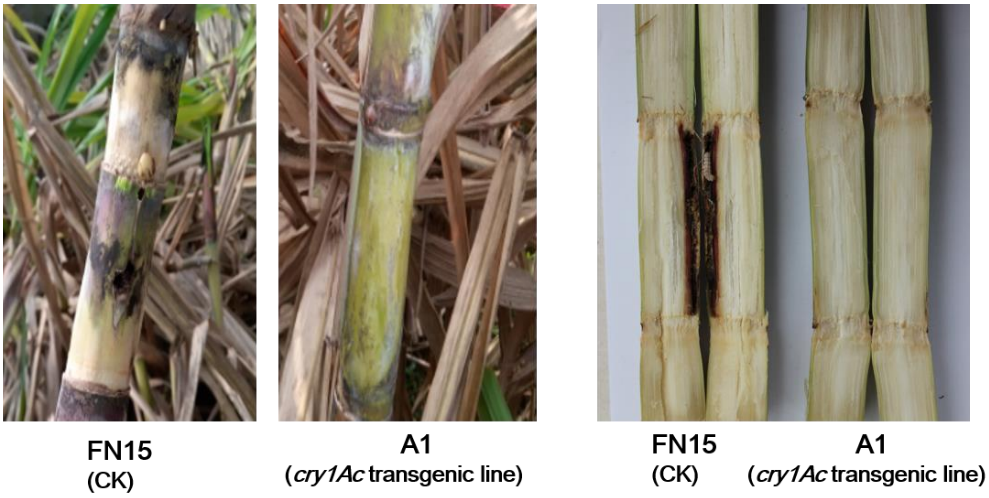

Figure S1 Insect-resistant phenotype of the *cry1Ac* sugarcane and the receptor variety FN15 (CK).
