# Supplementary material for: Foreign cry1Ac gene integration and endogenous borer stress-related genes synergistically improve insect resistance in sugarcane
Source: BMC Plant Biol. 2018 Dec 10;18:342. doi: 10.1186/s12870-018-1536-6 (PMC6288918; doi:10.1186/s12870-018-1536-6)
Supplement: Supplementary file 2 — Supplementary RNA-Seq data file, including Table S1, Table S2, Table S3, Figure S2 and Figure S3 Table S1. The base pair information of high quality clean reads for de novo assembly. Table S2. Assembly statistics of the sugarcane unigenes. Table S3. Unigenes basic annotation statistics with Nr, Swiss-Prot, KOG and KEGG. Figure S2. The length distribution of sugarcane unigenes. Figure S3. Statistics of differentially expressed genes. (PDF 56 kb) [file 12870_2018_1536_MOESM2_ESM.pdf]

Table S1 The base pair information of high quality clean reads for *de novo* assembly

Sample	Data After filter (bp)	Q20 value	Q30 value	GC content
CK-1	6,743,334,750	6,481,065,180 (96.11%)	6,194,777,687 (91.87%)	56.48%
CK-2	6,801,237,250	6,550,527,142 (96.31%)	6,275,290,345 (92.27%)	56.39%
CK-3	7,972,541,750	7,624,427,292 (95.63%)	7,253,940,425 (90.99%)	56.25%
A1-1	6,784,743,500	6,495,169,136 (95.73%)	6,185,428,811 (91.17%)	56.27%
A1-2	7,158,814,250	6,861,145,561 (95.84%)	6,539,770,262 (91.35%)	56.38%
A1-3	6,509,171,750	6,221,441,131 (95.58%)	5,914,658,737 (90.87%)	55.74%
A5-1	7,457,190,250	7,296,652,360 (97.85%)	7,111,436,672 (95.36%)	55.51%
A5-2	7,087,939,750	6,767,513,158 (95.48%)	6,426,802,683 (90.67%)	55.88%
A5-3	6,104,132,000	5,853,426,582 (95.89%)	5,584,588,467 (91.49%)	55.40%
B4-1	7,742,686,000	7,589,666,777 (98.02%)	7,412,607,738 (95.74%)	55.41%
B4-2	6,268,547,250	6,056,979,622 (96.62%)	5,822,729,281 (92.89%)	56.40%
B4-3	6,871,830,250	6,632,380,640 (96.52%)	6,369,021,045 (92.68%)	55.92%

Table S2 Assembly statistics of the sugarcane unigenes

Assembly statistics	Number and characteristics of unigenes
Total number of unigenes	65,995
GC percentage%	51.99%
N50	1,049
Max length	16,441
Min length	230
Average length	715.14

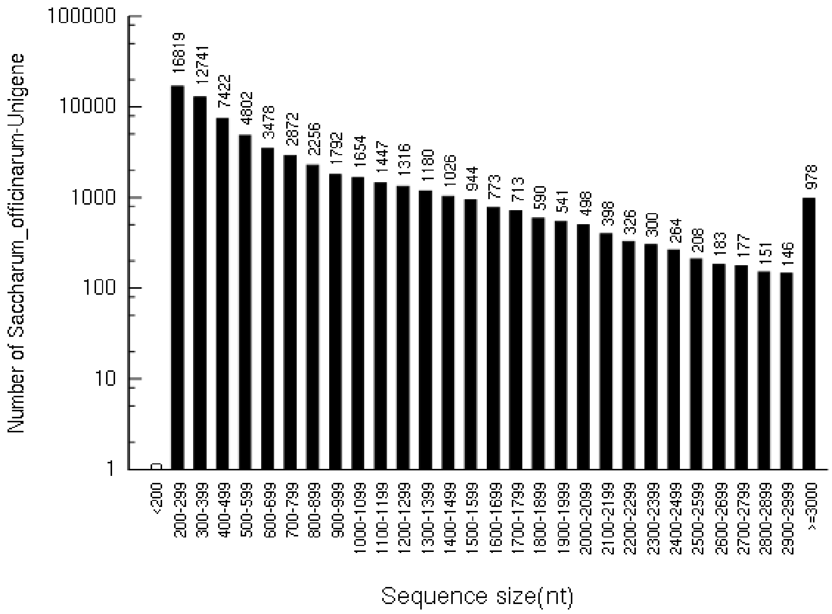

Figure S2 The length distributionof sugarcane unigenes

Table S3 Unigenes basic annotation statistics with Nr, Swissprot, KOG and Kegg

Unigenes basic annotation statistics	Statistic results
Total Unigenes	65,995
Nr	41,193
Swissprot	24,190
KOG	20,709
KEGG	9,092
Annotation genes	41,262
Without annotation gene	24,733

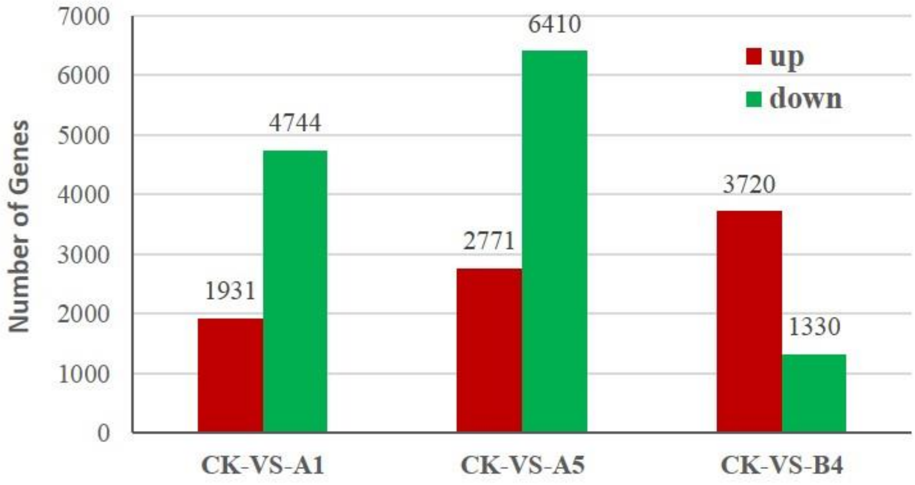

Figure S3 Statistics of differentially expressed genes
