# Supplementary figures and images for: Foreign cry1Ac gene integration and endogenous borer stress-related genes synergistically improve insect resistance in sugarcane
Source: BMC Plant Biol. 2018 Dec 10;18:342. doi: 10.1186/s12870-018-1536-6 (PMC6288918; doi:10.1186/s12870-018-1536-6)

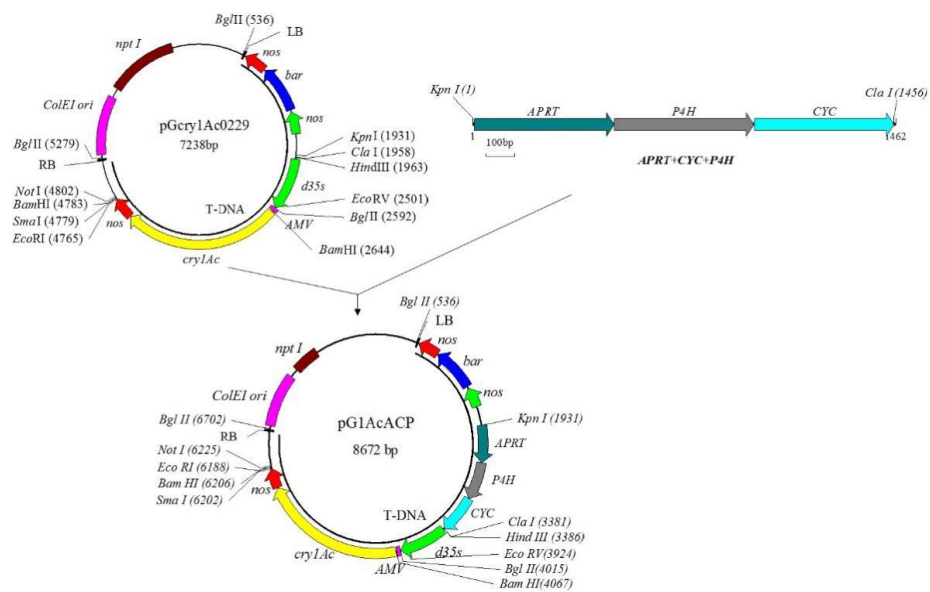

**Figure. S4 Construction of the plasmid pG1AcAPC (p1AcAPC)**

Supplement: Supplementary file 4 — Figure S4. Construction of the plasmid pG1AcAPC (p1AcAPC). (PDF 148 kb) [file 12870_2018_1536_MOESM4_ESM.pdf]
