# Supplementary material for: Foreign cry1Ac gene integration and endogenous borer stress-related genes synergistically improve insect resistance in sugarcane
Source: BMC Plant Biol. 2018 Dec 10;18:342. doi: 10.1186/s12870-018-1536-6 (PMC6288918; doi:10.1186/s12870-018-1536-6)
Supplement: Supplementary file 5 — Table S5. The primer sequences and TaqMan probe sequences for real-time PCR. (DOCX 12 kb) [file 12870_2018_1536_MOESM5_ESM.docx]

**Additional file 4**

**Table S5 Primer sequences and TaqMan probe sequence for real-time PCR**

| **Primer and Probe name** | **Primer sequence and TaqMan probe sequence** |
| --- | --- |
| *cry1Ac*-qF | 5’-ACCGGTTACTCCCATCGA-3’ |
| *cry1Ac*-qR | 5’-CCAGCACCTGGCACGAA-3’ |
| *cry1Ac*-Probe | 5’-FAMTCTCCTTGTCCTTGACACAGTTTCTGCTCA TAMRA-3’ |
| *APRT*-qF | 5’-AGGGAAGTGGTTCGGTGATG-3’ |
| *APRT*-qR | 5’-TGATAAAGAGCACATGAACCAACA-3’ |
| *APRT*-Probe | 5’-FAMTGTCAGTGGAAAAACCCGGTCACCATAMRA-3’ |
| *P4H*-qF | 5’-GTGAAAATATAGTAAAAACTGCAAAGGA-3’ |
| *P4H*-qR | 5’-TTGTACTCTCCGCGGTTTCTC-3’ |
| *P4H*-Probe | 5’-FAMTGAAGCCATCAACATTAGCCCTGAGGATAMRA-3’ |
| *CYC*-qF | 5’-CAGATATGGAAAGCACAAAAATGACT-3’ |
| *CYC*-qR | 5’-GCTTACCAAGGGCTAAGTAATGTCA-3’ |
| *CYC*-Probe | 5’-FAMATGACATTCCCTTGCCTATGCTGTCGGTAMRA-3’ |
